# 4-Coumarate CoA Ligase Family in Soybean Responds to *Heterodera glycines*

**DOI:** 10.3390/cimb47100795

**Published:** 2025-09-25

**Authors:** Hui Wang, Shumei Liu, Shunbin Zhang, Fengjiao Fan, Chuanwen Yang, Yuxi Duan, Qiumin Chen, Chen Liu

**Affiliations:** 1 College of Bioscience and Biotechnology, Shenyang Agricultural University, Shenyang 110866, China; 2 Plant Protection College, Shenyang Agricultural University, Shenyang 110866, China

**Keywords:** soybean cyst nematode, cell wall, lignin, 4-coumarate coenzyme A ligase

## Abstract

Soybean cyst nematode (SCN) development depends on syncytium formation, which requires cell-wall degradation and fusion. Lignin, the main barrier in cell walls, is critical for SCN resistance. 4-Coumarate: CoA ligase (4CL) drives the phenylpropanoid pathway by converting p-coumaric acid to p-coumaroyl-CoA, supplying lignin precursors. Here, resistant cv. Huipizhiheidou accumulated more lignin than susceptible Williams 82 after SCN inoculation. SCN stress induced distinct *Gm4CL*-family expression profiles across cultivars; *Gm4CL3* and *Gm4CL4* were markedly upregulated in Huipizhiheidou. Transient expression of *Gm4CL3* in tobacco thickened leaf cell walls, implying enhanced wall reinforcement against SCN. Thus, 4CLs, especially *Gm4CL3*, may promote lignin deposition and secondary wall thickening to strengthen soybean SCN resistance.

## 1. Introduction

Soybean cyst nematode (SCN, *Heterodera glycines* Ichinohe), a soil-borne obligate parasite, inflicts severe annual yield losses worldwide. The nematode’s life cycle unfolds in three phases—egg, juvenile, and adult—of which the second-stage juvenile (J2) is the sole infectious form. Upon sensing host-derived root exudates [1], J2s migrate toward the root, deploy secreted stylet enzymes to breach the epidermis, and inflict mechanical injury. They then navigate through the cortex by enzymatically remodeling and fusing plant cell walls before establishing a multinucleate syncytium that serves as a permanent feeding site [2]. Riggs (1973) first proposed that thickened or modified cell walls could impede nematode ingress and syncytium formation, underscoring an intimate link between cell-wall-mediated defenses and the success of SCN colonization [3].

Cell-wall resistance is inseparable from the chemical composition of the cell wall. The lignin in the secondary cell wall constitutes the cell wall resistance and is one of the components of plant lignification to respond to adversity stress. Lignin is likely to be an important component in regulating SCN resistance [4,5]. Lignin is the second-largest polymer in the world after cellulose [6,7], and it is involved in regulating plant growth and development and stress response [8] and is a component that constitutes defense structures such as cell wall resistance, casparian strip, and endodermis. By analyzing the biosynthesis of lignin, it is found that lignin biosynthesis is mainly in three stages: the shikimic acid metabolic pathway, phenylpropane metabolic pathway, and lignin synthesis specific pathway; shikimic acid and its downstream metabolic pathways are key regulatory pathways for plant secondary metabolism. In the process of lignin synthesis, 4CL is a key enzyme connecting phenylpropane metabolism and the lignin synthesis pathway. Studies have shown that 4CL is involved in regulating the biosynthesis of lignin and secondary cell walls [9], which helps plants to resist adversity [10].

4CL occurs as a multigene family whose individual members channel distinct branches of phenylpropanoid metabolism [11,12,13]. Acting immediately downstream of PAL and C4H, 4CL catalyzes the ATP-dependent activation of p-coumaric and related hydroxycinnamic acids into their corresponding CoA thio-esters, thereby committing carbon flux toward lignin biosynthesis [11]. Owing to this pivotal position, 4CL has been cloned and functionally characterized in Arabidopsis [13], soybean [14], maize [15], switchgrass [16], tobacco [17], Manchurian ash [9], and black pepper [18]. However, the transcriptional dynamics and functional relevance of 4CL under SCN stress remain unexplored.

Soybean (*Glycine max*) is a leading global cash crop and the foremost source of plant protein and oil, yet its productivity is sharply curtailed by SCN. Breeding resistant cultivars have therefore become a research priority. 4CL belongs to the adenylate-forming enzyme superfamily. Soybean contains four 4CL paralogs [15]. Here, we show that SCN challenge triggers pronounced lignin accumulation in the roots of Huipizhiheidou (ZDD2315), accompanied by significant upregulation of the 4CL gene family, particularly *Gm4CL3* (Glyma.11g010500) and *Gm4CL4* (Glyma.01g232400), in resistant lines (ZDD2315, ZDD7170 and ZDD1412) under SCN3 stress and transient over-expression of *Gm4CL3* in tobacco thickened the cell wall, demonstrating that *Gm4CL3* reinforces physical barriers. Collectively, our findings illuminate the pivotal role of *Gm4CL* genes in bolstering soybean SCN resistance and provide a robust foundation for their functional exploitation in breeding programs.

## 2. Materials and Methods

### 2.1. Plant and Nematode

A soybean susceptible variety, Williams 82, and three resistant soybean varieties Huipizhiheidou (ZDD2315), Harbinxiaoheidou (ZDD7170), Xiaoliheidou (ZDD1412) were grown at 27 °C with a 12 h photoperiod in a greenhouse. Soybean cyst nematode (*Heterodera glycines* Ichnohe, SCN) race 3, one of the most widely distributed races in China, was tested in this study. The suspensions of second-stage juveniles (J2s) were concentrated to 400 pcs/mL through a 23 μm sieve and then mixed 1:1 with a sterilized 0.2% water-agar to 200 pcs/mL (The final concentration of water–agar working solution is 0.1%). Each plant received 5 mL of this suspension (≈1000 J2) poured directly around the root zone. SCN infested soil was mixed 1:1 with sterilized fine sand. Soybean seeds were sown to make SCNs grow and multiply. The cultivation lasted for two months, SCNs were separated from the infested soil, and the eggs were collected and incubated in Baermann Funnels at 27 °C, avoiding light. The hatched second-stage juveniles (J2s) were collected daily, and new distilled water was added. In order to ensure the activity of J2s, the collection process will be completed within one week. The isolated J2s SCN were utilized to infect various soybean materials.

### 2.2. Lignin Dyeing

Lignin changes before and after inoculation were assessed via phloroglucinol staining. Select soybean roots 0, 10, and 15 days post-SCN inoculation; soak the sample in the pre-configured fixative solution (95% ethanol:glacial acetic acid (*v*/*v*) = 1:1) for 24 h; rinse the fixative solution with distilled water; then put it in a saturated chloral aqueous solution (100 g chloral hydrate dissolved in 50 mL ddH_2_O), vacuum-treat it for 10 min, and let it dry until it is transparent at room temperature. Soak the root tissue with 1% phloroglucinol solution (5 g of phloroglucinol, 25 mL of 95% ethanol, ddH_2_O to a final volume of 500 mL) for 2–5 min, add a few drops of concentrated hydrochloric acid and immediately photograph the sample under an ordinary light microscope (DP80; Olympus Corporation, Tokyo, Japan).

### 2.3. Quantitative RT-PCR

Total RNA was isolated from soybeans roots using the Total RNA Extraction Reagent (Vazyme, Nanjing, China); first-strand cDNA was synthesized using the PrimeScript™ RT reagent kit (TaKaRa, Beijing, China). qRT-PCR was performed to detect gene transcript levels using the 2X Universal SYBR Green Fast qPCR Mix (ABclonal, Wuhan, China) on a CFX96 qRT-PCR detection system (Bio-rad, San Francisco, CA, USA). Data were analyzed using the 2^−ΔΔCq^ method. Three groups of roots were sampled. *Tubulin* (Glyma. 15G132200) as an internal reference gene. All primers used for qRT-PCR are listed in Table S1. Each data point was replicated three times.

### 2.4. Vector Constructs and Plant Transformation

Full-length CDS of *Gm4CL3* and *Gm4CL4* (downloaded from Phytozome, https://phytozome-next.jgi.doe.gov/, accessed on 18 October 2019) were PCR-amplified with NcoI–SpeI-tailed primers and seamlessly inserted into NcoI/SpeI-linearized pCAMBIA1302-GFP via In-Fusion cloning. The resulting constructs were transiently expressed in *Nicotiana benthamiana* leaves by *Agrobacterium*-mediated infiltration, with empty pCAMBIA1302-GFP serving as the control. Target-gene expression was monitored by qRT-PCR, and transmission electron microscopy (HT7800 TEM; Hitachi Ltd., Tokyo, Japan) was employed to evaluate the impact of 4CL gene on cell-wall architecture. All primers are listed in Table S1.

### 2.5. Statistical Analysis

The MEGAX and GeneDoc software were used for multiple sequence alignment and phylogenetic analysis. Multiple sequence alignments of the full-length 4CL amino-acid sequences were generated with MEGA X (v11.0.13) using the built-in ClustalW algorithm with default parameters. Ambiguously aligned regions were manually inspected and trimmed in GeneDoc (v2.7.000). For the phylogenetic tree, the cleaned alignment was subjected to maximum-likelihood (ML) analysis in MEGA X. Protein Graphpad Prism 9.0 and Microsoft Excel 2019 were used for data statistics and graph analysis. Comparisons between two groups were conducted using Student’s *t*-test. Significance levels are denoted as follows: * *p* < 0.05, ** *p* < 0.01, *** *p* < 0.001, and **** *p* < 0.0001. All values are presented as means ± standard deviation (SD) from at least three biological replicates.

## 3. Results

### 3.1. Lignin Response in Roots of SCN-Resistant and SCN-Susceptible Soybean Varieties

**Figure 1:**
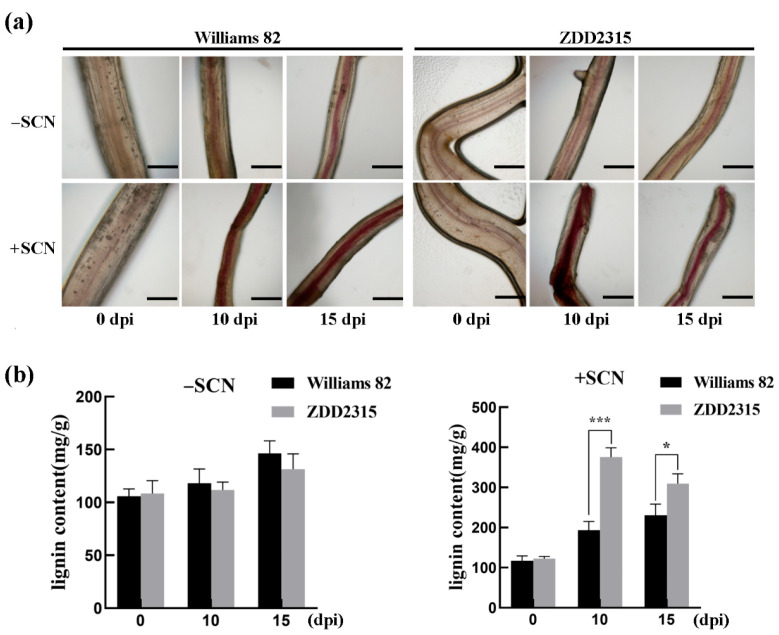
Lignin accumulation in Williams 82 and ZDD2315 roots following SCN3 inoculation. (**a**) Representative images depict phloroglucinol-HCl staining of lignin in roots sampled at 0, 10, and 15 days post-inoculation (dpi). Bar: 1 cm. (**b**) Measurement of lignin content in soybean roots before and after SCN inoculation. Asterisks denote statistical significance: * *p* < 0.05, *** *p* < 0.001. Data represent three biological replicates.

To dissect the mechanisms governing soybean resistance to SCN, we compared the susceptible cultivar Williams 82 with the resistant cultivar ZDD2315. The root cell wall constitutes the first physical barrier, and lignin is its key load-bearing component. Lignin staining at 10 days post-inoculation revealed pronounced lignin accumulation in ZDD2315 roots, whereas levels in Williams 82 remained unchanged (Figure 1a). During the ~10 d required for syncytium establishment, the resistant host markedly increases lignin deposition to impede SCN development.

Additional measurements of lignin content across different lines revealed no significant difference between the Williams 82 and ZDD2315 under non-inoculated conditions. However, following SCN inoculation, lignin content in ZDD2315 roots was significantly higher than that in Williams 82 (Figure 1b). The results suggest that under SCN stress, the ZDD2315 variety can enhance lignin synthesis to combat stress, whereas Williams 82 can only synthesize a small amount of lignin, showing greater sensitivity to SCN.

### 3.2. Bioinformatics Analysis of 4CL Family Genes

**Figure 2:**
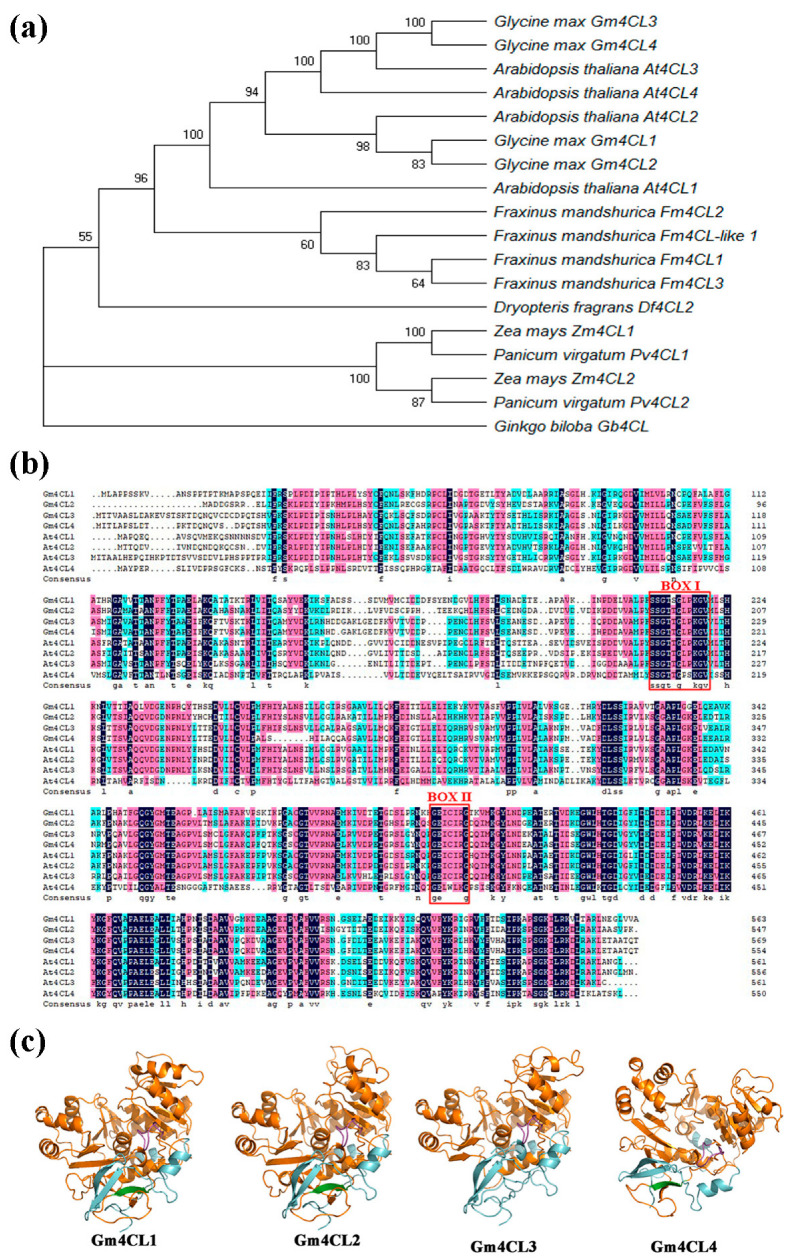
Sequence analysis of 4CL. (**a**) Phylogenetic tree of the 4CL gene family. Branches indicate evolutionary relationships among Gm4CL1 (Glyma.17g064600), Gm4CL2 (Glyma.13g372000), Gm4CL3 (Glyma.11g010500), and Gm4CL4 (Glyma.01g232400) from *Glycine max*; At4CL1 (AT1G51680), At4CL2 (AT3G21240), At4CL3 (AT1G65060), and At4CL4 (AT1G20500) from *Arabidopsis thaliana*; Zm4CL1 (NM_001111788) and Zm4CL2 (NM_001156842) from *Zea mays*; Pv4CL1 (EU491511.1) and Pv4CL2 (JF414903.1) from *Panicum virgatum*; Fm4CL1 (KJ531400.1), Fm4CL2 (KJ531401.1), Fm4CL3 (KJ531402.1), and Fm4CL-like1 (KJ531403.1) from *Fraxinus mandshurica*; Df4CL2 (KF836752.1) from *Dryopteris fragrans*; Gb4CL (KU820947.1) from *Ginkgo biloba*. (**b**) Multiple amino-acid alignment of soybean 4CL paralogs (Gm4CL1-4) and *Arabidopsis* 4CL (At4CL1-4). Red boxes highlight the conserved BOX I (SSGTTGLPKGV) and BOX II (GEICIRG) motifs. (**c**) Homology modeling: Four Gm4CL isoforms were modeled with SWISS-MODEL using Arabidopsis At4CL (PDB: 3tsy.1) as the template. The conserved BOX I (pink) and BOX II (green) motifs are highlighted.

Although family members possess overlapping functions, their contributions are not identical. In *Arabidopsis*, At4CL1, At4CL2, At4CL3, and At4CL4 participate mainly in flavonoid and lignin metabolism. A phylogenetic tree constructed from *Arabidopsis*, soybean, maize, switchgrass, tobacco, Manchurian ash, and ginkgo revealed a high conservation of 4CL proteins across species. Gm4CL3 and Gm4CL4 cluster with At4CL3/4CL4, whereas Gm4CL1 (Glyma.17g064600) and Gm4CL2 (Glyma.13g372000) are closer to At4CL2 (Figure 2a). Whether these soybean genes differentially regulate flavonoid versus lignin pathways in SCN-stressed roots remains to be tested.

Multiple sequence alignments generated with MEGA X and refined in GeneDoc revealed two hallmark motifs that define the 4CL families of soybean and *Arabidopsis*: BOX I (SSGTTGLPKGV), the canonical ATP-binding signature of adenylate-forming enzymes, and BOX II (GEICIRG), a motif unique to 4CL proteins [19] (Figure 2b). The strict conservation of these motifs across Gm4CL1–4 confirms that the soybean 4CL clade belongs to the acyl-activating enzyme superfamily and constitutes a key regulatory node in lignin biosynthesis and phenylpropanoid metabolism. Notably, the pairwise sequence identity between soybean and *Arabidopsis* 4CL proteins exceeds 67% (Figure 2b). Homology modeling (At4CL template; SWISS-MODEL) showed all Gm4CLs share a conserved catalytic core (BOX I and II), with Gm4CL3 BOX II deviating suggestive of functional divergence (Figure 2c). N-terminal extensions, pocket micro-substitutions, and surface-loop indels provide structural footholds for isoform-specific regulation, substrate preference, and protein–protein interactions, implying functional conservation between the two species.

### 3.3. Response of the Gm4CL Gene Family to SCN Stress

**Figure 3:**
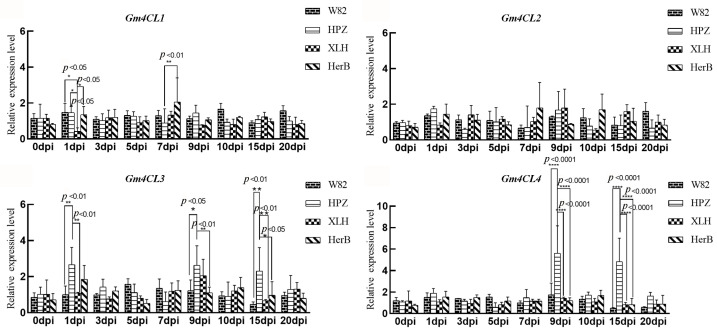
Relative expression of *Gm4CL* genes under SCN stress in four soybean cultivars: susceptible Williams 82 (W82), resistant ZDD2315 (HPZ), moderately resistant ZDD7170 (HerB), and moderately resistant ZDD1412 (XLH). Data are presented as means ± SD. Asterisks indicate statistical significance: * (*p* < 0.05), ** (*p* < 0.01), and **** (*p* < 0.0001).

To systematically evaluate the dynamic contribution of the 4CL gene family to soybean defense against SCN, we conducted a time-course expression analysis of *Gm4CL1–Gm4CL4* in four representative genotypes: the resistant cultivar ZDD2315/HPZ, the susceptible cultivar Williams 82, and two intermediate lines (ZDD1412/XLH and ZDD7170/HerB). Following SCN3 inoculation, *Gm4CL1* and *Gm4CL2* showed no significant differences among the four cultivars in root tissues from 0 to 20 dpi (Figure 3). This suggests that these two genes are unlikely to be directly involved in root-mediated SCN resistance.

In contrast, *Gm4CL3* and *Gm4CL4* exhibited pronounced and sustained differential expressions between resistant and susceptible genotypes. Specifically, *Gm4CL3* transcripts in resistant HPZ roots were already 2.7-fold higher than in Williams 82 at 1 dpi and remained elevated by 2.6-fold and 2.3-fold at 9 and 15 dpi, respectively (Figure 3). *Gm4CL4* showed an even more robust induction, reaching 5.6-fold and 4.0-fold higher levels in HPZ relative to Williams 82 at 9 and 15 dpi, respectively (Figure 3). These data demonstrate that *Gm4CL3* and *Gm4CL4* are specifically upregulated in resistant soybean at critical developmental stages post-inoculation, implicating these two genes as central players in SCN tolerance. Their precise roles warrant further functional validation and mechanistic investigation.

### 3.4. Expression of 4CL-Downstream Lignin-Peroxidase (POD) Genes Under SCN Stress

**Figure 4:**
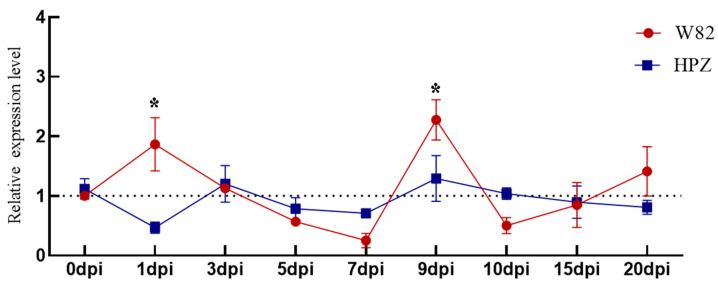
Relative expression of *GmPOD53* under SCN stress in susceptible Williams 82 (W82), and resistant ZDD2315 (HPZ) soybean cultivars. Data are presented as means ± SD. Asterisks indicate statistical significance: * (*p* < 0.05).

Peroxidase (POD) is a core component of the plant defense arsenal and simultaneously modulates lignin turnover. Time-course qPCR revealed markedly different *GmPOD53* (Glyma.02G171600.1) dynamics in resistant HPZ versus susceptible Williams 82 after SCN3 inoculation. In HPZ, *GmPOD53* transcripts remained stable throughout the 15-d assay (Figure 4), consistent with the early and sustained upregulation of *Gm4CL3/4* that restricts SCN ingress and thereby minimizes lignin bursts. In Williams 82, *GmPOD53* expression declined from 1 to 7 dpi, followed by a pronounced surge at 9 dpi that surpassed the levels observed in HPZ (Figure 4). These data indicate that robust 4CL-mediated lignification in HPZ curtails SCN3 advance, resulting in only a muted POD response, whereas the susceptible cultivar exhibits reactive POD bursts that coincide with irregular lignin deposition and reduced SCN resistance.

### 3.5. Over-Expression of Gm4CL3 Promotes Cell-Wall Thickening

**Figure 5:**
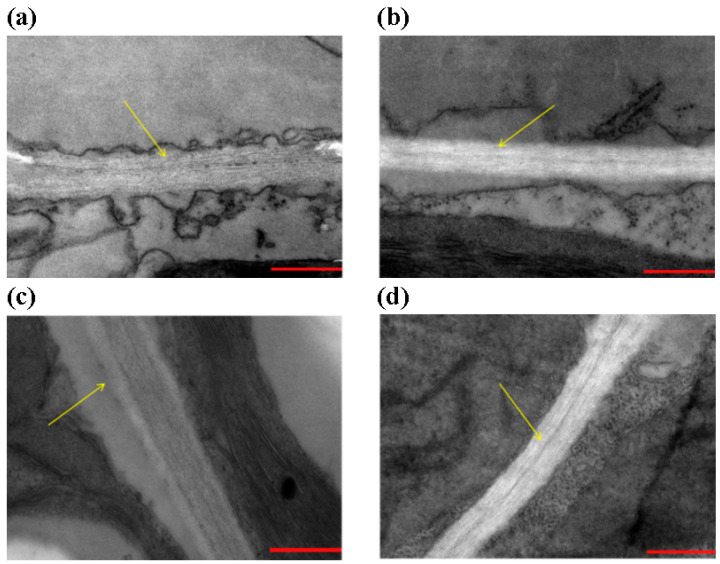
Effect of Gm4CL over-expression on cell-wall thickness. Transmission electron microscopy images of *Nicotiana benthamiana* leaves transiently transformed with Gm4CL1–4 constructs. Scale bar = 500 nm; magnification 15 k× (Hitachi TEM). (**a**) PCAMBIA1302-GFP empty vector, (**b**) suspension buffer control, (**c**) PCAMBIA1302-GFP-Gm4CL3, and (**d**) PCAMBIA1302-GFP-Gm4CL4, respectively. Yellow arrow represents secondary cell wall.

Lignin is a key structural component of the cell wall, which constitutes the first physical barrier against pathogen ingress. Tobacco leaves transiently expressing *Gm4CL3* were examined by transmission electron microscopy. While suspension, empty-vector, and *Gm4CL4* constructs did not alter wall thickness (Figure 5a,b,d), *Gm4CL3*-overexpressing tobacco leaves displayed a visible wall-thickening phenotype (Figure 5c), suggesting that *Gm4CL3* directly promotes wall lignification. Collectively, these data support the hypothesis that 4CL enhances SCN resistance through cell-wall reinforcement, although the precise regulatory network remains to be elucidated.

## 4. Discussion

Soybean cyst nematode (SCN) inflicts enormous direct and indirect losses on global soybean production every year. The root’s barrier system—of which lignin is a central component—acts as the first line of defense, repairing mechanical injury and reinforcing cell-wall resistance both early and late in infection [20]. Consequently, lignin biosynthesis is intimately tied to the plant’s response to SCN. 4-Coumarate: CoA ligase (4CL), the pivotal enzyme channeling phenylpropanoid flux into lignin, is encoded by a small gene family whose members serve distinct metabolic routes. In *Arabidopsis*, *At4CL1* is the primary driver of lignin deposition; *At4CL2*, *At4CL3*, and *At4CL4* contribute only when *At4CL1* is compromised [13,21]. In soybean, *Gm4CL3* emerges as the principal contributor, likely because additional, SCN-specific signaling pathways converge on *Gm4CL3*, amplifying its role in SCN resistance and endowing it with superior SCN tolerance in this study. Soybean possesses four 4CL paralogs (Gm4CL1–4) [15]. Based on the genome of *Arabidopsis*, 20 Gm4CL gene family subsets were screened, with expression mainly distributed in cytoplasmic peroxisomes, and showing homology to the *4CL* genes of peanut (*Arachis hypogaea*) and *Arabidopsis* [22]. Phylogenetic analysis places Gm4CL3 and Gm4CL4 in one clade; their markedly higher expression in the resistant cultivar HPZ, compared with Williams 82 and two other resistant lines, suggests they may confer SCN resistance by promoting lignin biosynthesis and cell-wall fortification. Whether other 4CL members—or distinct monolignol combinations—also contribute remains to be tested.

Beyond SCN, 4CL-mediated CoA-thioester formation influences plant growth and abiotic stress tolerance. Over-expression of *Fm4CL-like1* in tobacco elevated lignin by 39.5% and improved drought tolerance [9], while salt stress selectively upregulated *4CL2*, *4CL11*, and *4CL12*. It is well-known fact that 4CL is important for soybean reaction to nematodes [23]. To dissect 4CL function under SCN stress, we inoculated four cultivars of contrasting resistance with SCN3. Only *Gm4CL3* and *Gm4CL4* exhibited significant, time-specific induction in the resistant cultivar HPZ at 9 and 15 dpi—stages that coincide with SCN mechanical wounding and syncytium maturation. Notably, *Gm4CL3* transcripts rose sharply as early as 1 dpi, suggesting a role in the initial perception of nematode attack. Concurrent upregulation of *Gm4CL3* and the *GmPOD53* gene at both 1 and 9 dpi further implies that *Gm4CL3* is more closely associated with the SCN infection process. *Gm4CL3* and *Gm4CL4* transcripts continued to accumulate on days 9 and 15, whereas no significant change was observed on day 10. Such temporal dynamics suggest that *Gm4CL3* may synergize with other resistance loci to consolidate defense—either by reinforcing cell-wall barriers or by priming downstream defense cascades. Alternatively, *Gm4CL3* could act autonomously, for example, by modulating oxidative-stress responses that contribute to SCN resistance. Nevertheless, given the functional redundancy and organ specificity often observed within the 4CL family, we cannot exclude the possibility that *Gm4CL1* and *Gm4CL2* are induced in leaves, stems, or flowers and exert indirect effects on overall resistance through flavonoids or other secondary metabolites.

SCN deploys cell-wall-degrading enzymes and modifying proteins to weaken the wall and facilitate colonization [24]. Thus, the physical barrier provided by the cell wall is a key battleground. In Williams 82, *GmPOD53* transcript levels dropped from 1 to 7 dpi and then surged at 9 dpi, exceeding those in HPZ. This transient elevation aligns with the putative waves of SCN3 penetration and syncytium formation, triggering an ROS burst that demands robust POD-mediated detoxification. However, heightened POD activity simultaneously accelerates lignin depolymerization, undermining cell-wall integrity and rendering roots increasingly vulnerable to subsequent nematode ingress. Using tobacco as a transient expression system, we observed that *Gm4CL3* over-expression visibly thickened cell walls, providing preliminary evidence that 4CL-mediated reinforcement can impede SCN ingress. However, Gm4CL4 failed to alter lignin deposition in tobacco, lignin biosynthesis is highly complex; distinct 4CL members may operate under divergent signaling cues and interact with different partners. Definitive confirmation awaits stable soybean transformation and mechanistic dissection. Because 4CL isoforms differ in substrate preference and the monolignols they ultimately produce, it remains unclear whether SCN resistance arises from a single monomer or from synergistic interactions among several. Control of SCN is therefore a dynamic process: multi-angle comparisons, continuous methodological innovation, and systematic resistance strategies will be essential for future success.

## 5. Conclusions

The present study provides compelling evidence that the resistant soybean cultivar HPZ mounts a robust lignin-based defense when challenged by SCN. Comprehensive analysis of the four highly conserved soybean 4CL paralogs (Gm4CL1–4) revealed that only *Gm4CL3* and *Gm4CL4* were transcriptionally activated in HPZ roots. Downstream *POD* gene, responsible for both lignin polymerization and turnover, remained stable in HPZ roots, indicating that lignin synthesis rather than degradation prevailed under SCN stress. Conversely, *POD* transcripts in Williams 82 fluctuated dramatically, coinciding with nematode penetration waves and likely reflecting a futile attempt to counteract SCN-induced oxidative bursts. Transient over-expression of *Gm4CL3* in tobacco leaves resulted in a measurable increase in cell-wall thickness, providing in vivo evidence that 4CL-mediated lignin deposition reinforces the physical barrier against nematode ingress. Our findings establish a model in which SCN perception triggers rapid upregulation of *Gm4CL3* and *Gm4CL4* in resistant soybean, leading to enhanced lignin biosynthesis, thickened cell walls, and ultimately, improved whole-plant resistance.

## Data Availability

Data will be made available on request.

## Supplementary Materials

The Supplementary Material for this article can be found online. The following supporting information can be downloaded at https://www.mdpi.com/article/10.3390/cimb47100795/s1.
